# Correction: Hyperosmotic stress memory in Arabidopsis is mediated by distinct epigenetically labile sites in the genome and is restricted in the male germline by DNA glycosylase activity

**DOI:** 10.7554/eLife.44302

**Published:** 2018-12-21

**Authors:** Anjar Wibowo, Claude Becker, Gianpiero Marconi, Julius Durr, Jonathan Price, Jörg Hagmann, Ranjith Papareddy, Hadi Putra, Jorge Kageyama, Jorg Becker, Detlef Weigel, Jose Gutierrez-Marcos

Wibowo A, Becker C, Marconi G, Durr J, Price J, Hagmann J, Papareddy R, Putra H, Kageyama J, Becker J, Weigel D, Gutierrez-Marcos J. 2016. Hyperosmotic stress memory in Arabidopsis is mediated by distinct epigenetically labile sites in the genome and is restricted in the male germline by DNA glycosylase activity. *eLife* **5**:e13546. doi: 10.7554/eLife.13546.

Published 31, May 2016

There was a mistake in the Figure Legend for Figure 1C and 1D - the concentration of NaCl used in the experiment was 150 mM NaCl and not 200 mM NaCl as previously stated in the text. We discovered the mistake when Dr Hadi Putra (co-author of the paper) used the same assay for another project. Below is the corrected text:

(**C**) Survival of P1 and P2 seedlings grown on medium with **150 mM NaCl**. At least 300 seedlings tested per triplicate in two independent experiments. Asterisks indicate a significant difference between the control group of the same generation (unpaired Student’s t-test; * p<0.05, ** p<0.01, ns p>0.05). Horizontal bar corresponds to median, whiskers indicate entire 95th percentile.

(**D**) Survival of wild-type (wt) and RdDM and DNA methylation mutant P1 and P2 seedlings on medium with **150 mM NaCl** (unpaired Student’s t-test; * p<0.05, ns p>0.05). Error bars indicate standard deviation.

The article has been updated accordingly.

